# The impact of the limbal niche interactions on the self-renewal capability of limbal epithelial stem cells

**DOI:** 10.3389/fcell.2025.1667309

**Published:** 2025-10-29

**Authors:** Sara Aghazadeh, Qiuyue Peng, Fereshteh Dardmeh, Jesper Østergaard Hjortdal, Vladimir Zachar, Hiva Alipour

**Affiliations:** ^1^ Regenerative Medicine, Department of Health Science and Technology, Aalborg University, Aalborg, Denmark; ^2^ Department of Ophthalmology, Aarhus University Hospital, Aarhus, Denmark

**Keywords:** limbal stem cells, conditioned media, fibronectin, stemness, *PEDF*, *HES1*

## Abstract

**Introduction:**

The corneal homeostasis is maintained by limbal epithelial stem cells (LESCs), which reside in the limbal niche. This microenvironment comprises the cells, the extracellular matrix (ECM), and their interactions that balance the quiescent and proliferative states of LESCs. The stress caused by removing the cells from their niche triggers the quiescent stem cells to enter the proliferative state, which is beneficial for *in vitro* expansion, but reduces their self-renewal capability, making them less suitable for transplantation. Fibronectin (FN), a key ECM component, widely used in tissue engineering and scaffold structure, has been shown to preserve the self-renewal ability of LESCs *in vitro*. In parallel, paracrine growth factors are crucial for maintaining limbal niche homeostasis and promoting corneal epithelial regeneration. Limbal-niche-cells-conditioned media is a potential reservoir of limbal niche paracrine growth factors. However, whether utilizing fibronectin and limbal-niche-cells-conditioned media can sustain or enhance the stemness and proliferation ability of LESCs *in vitro* has not yet been investigated.

**Methods:**

Primary cultures of limbal niche cells, including LESCs, limbal mesenchymal stromal cells (LMSCs), and limbal melanocytes (LM), were established from remnant human corneal transplant specimens, and human epidermal melanocytes (HEMn) were included as a negative control. The proliferation ability (doubling time) and self-renewal potential (as assessed by *PEDF* and *HES1* gene expressions) of LESCs were evaluated after culture in LM-, LMSC-, and HEMn-conditioned media, as well as coating with 3, 5, and 8 µg/cm^2^ concentrations of FN.

**Results:**

Compared to the control group, the LMSC- and LM-conditioned media showed a clear trend towards upregulated *PEDF* and *HES1* gene expressions. FN coating generally upregulated the expression of *PEDF* and *HES1* genes, with this effect being most prominent at 3 µg/cm^2^.

**Conclusion:**

These findings illustrate the potential of utilizing niche-cell-conditioned media and direct contact with FN on the self-renewal of LESCs *in vitro*. Further research is required to provide a more comprehensive understanding of these effects and to elucidate the underlying mechanisms of action.

## 1 Introduction

Corneal transparency is essential for vision and is maintained by the continuous regeneration of the corneal epithelium, a process sustained by local adult stem cells, the limbal epithelial stem cells (LESCs) (Ehlers and Hjortdal, 2005). Trauma, radiation, inflammation, autoimmune disorders, or prolonged contact lens use (Gonzalez et al., 2018; Le et al., 2018) can compromise the regenerative capacity of the LESCs, resulting in limbal stem cell deficiency (LSCD) and subsequent visual impairment (Le et al., 2018). Complications with current therapeutic approaches for LSCD, such as autologous serum administration (Azari and Rapuano, 2015), and allograft or autograft tissue transplantation (Cheung and Holland, 2017; Bilge, 2018), have led to increasing interest in *in-vitro* cultured cell transplantation as a promising alternative (Dobrowolski et al., 2015; Casaroli-Marano et al., 2015; Sacchetti et al., 2018). Cultivated epithelial stem cell transplantation (CLET) has emerged as a promising strategy for LSCD treatment (Sacchetti et al., 2018).

Similar to many other adult stem cells, LESCs typically reside in a quiescent, non-proliferative state, becoming activated only when required to restore tissue homeostasis (de Morree and Rando, 2023). Their ability to proliferate, self-renew, differentiate into mature cell types, and be expanded *in vitro* (Li and Clevers, 2010) makes them a great candidate for regenerative medicine (Li and Clevers, 2010; Ramalho-Santos and Willenbring, 2007). However, this advantage can quickly diminish when quiescent stem cells are cultured *in vitro*, presenting a significant challenge limiting the effectiveness of autologous transplantation therapies (Sacchetti et al., 2018; Marqués-Torrejón et al., 2021; Kobayashi et al., 2019; Quarta et al., 2016).

The concept of the stem cell niche, introduced by Schoefield et al., in 1978, highlighted the theory that the surrounding microenvironment regulates stemness and self-renewal, and removing stem cells from their niche leads to differentiation (Schofield, 1978). The quiescent state, enabling stem cells to support tissue regeneration in response to environmental signals (Urbán et al., 2019), is regulated by a combination of intrinsic and extrinsic mechanisms, including cell cycle and transcriptional regulators, metabolic factors, local and systemic signals, and interactions with the extracellular matrix (ECM) (Urbaìn and Cheung, 2021; Cho et al., 2019). In particular, cell-cell interactions regulate quiescence, self-renewal, differentiation, and survival (Farahzadi et al., 2023; Peerani and Zandstra, 2010; Pennings et al., 2018), while ECM proteins provide both mechanical scaffolding and biochemical signalling (Ferraro et al., 2010).

LESCs express various molecular markers, including P63, ABCG2, N-cadherin, NGF/Trk, integrin α9, integrin α6/CD71, HES1, nectin 3, and importin 13. PEDF is also recognized as a regulator of stemness, enhancing LESC self-renewal and proliferation. Moreover, HES1, as a key target gene of the Notch signalling pathway, is crucial for maintaining the LESC phenotype and quiescence (González et al., 2019; Kulkarni et al., 2010; Robertson et al., 2021).

Sacchetti et al. reported that less than 3% of isolated, cultivated, and transplanted LESCs are quiescent stem cells (P63+) capable of proliferation and renewal, necessitating repeated treatments (Sacchetti et al., 2018). This underscores the urgent need to develop strategies that enhance LESC self-renewal while maintaining the desirable transplantation characteristics.

The limbal niche includes both cellular and non-cellular components, including the extracellular matrix (ECM) and the niche cells (Mei et al., 2012; Polisetti et al., 2016), which provide regulatory signals crucial for LESC function (Mei et al., 2012; Polisetti et al., 2016; Mikhailova et al., 2015; Moreno et al., 2023).

The ECM contributes structural support and biochemical regulation, components like laminin (Polisetti et al., 2017), hyaluronan (HA) (Gesteira et al., 2017), and Fibronectin (FN) (Zheng et al., 2019), known to enhance LESC stemness. Niche-resident cells, including limbal melanocytes (LM), immune cells, LMSCs, vascular endothelial cells, and nerve cells, interact with LESCs either directly or through paracrine factors (Polisetti et al., 2022; Aghazadeh et al., 2024; Notara et al., 2010; Yazdanpanah et al., 2019; Polisetti et al., 2016).

LMs play a protective role against UV radiation, promote LESC stemness (Liu et al., 2018; Dziasko et al., 2015; Polisetti et al., 2021), and improve corneal regeneration (Yazdanpanah et al., 2019; Li et al., 2012; Polisetty et al., 2008; Dziasko and Daniels, 2016). Similar to other mesenchymal stem/stromal cells (MSCs), LMSCs secrete several growth factors, such as keratinocyte growth factor (KGF) (Gonzalez et al., 2018), nerve growth factor (NGF) (Amin et al., 2021), pigment epithelium-derived factor (PEDF) (Aghazadeh et al., 2024; Amin et al., 2021; Ho et al., 2013), insulin-like growth factor 1(IGF-1) (Trosan et al., 2012), fibroblast growth factor (FGF), ciliary neurotrophic factor, interleukin (IL)-1, and hepatocyte growth factor (HGF) (Amin et al., 2021), which are critical for preserving the limbal stem cell niche. While direct contact of LESCs with ECM components can further promote stemness (Polisetti et al., 2017; Zheng et al., 2019), the paracrine growth factor signalling also plays a key role in regulating LESCs’ stemness and niche homeostasis (Amin et al., 2021).

The interaction between cellular and non-cellular components in the limbal niche is essential for maintaining the stemness and self-renewal ability of limbal epithelial stem cells (LESCs). This importance is highlighted by the loss of these properties when quiescent LESCs are removed from their natural *in vivo* environment and cultured *in vitro* (Robertson et al., 2021). Nevertheless, several studies have indicated that ECM components and paracrine signalling can partially preserve the stemness and quiescent properties of LESCs (Urbaìn and Cheung, 2021; Robertson et al., 2021; Bonnet et al., 2021). Conditioned media (CM), which includes factors secreted by niche cells, has shown promise as a source of vital signals, although its role in supporting the self-renewal of LESCs *in vitro* is not yet fully explored (Jabbehdari et al., 2020a; Osugi et al., 2012; Smolinská et al., 2023). Therefore, to improve the potential of *in vitro* LESC culture for transplantation applications, this study aimed to systematically examine and compare the effects of fibronectin at concentrations of 3, 5, and 8 µg/cm^2^, along with conditioned media derived from LM, LMSC, and HEMn, on the proliferation (measured as doubling time) and stemness (assessed via *PEDF* and *HES1* expression) of LESCs *in vitro*.

## 2 Materials and methods

### 2.1 Cell isolation and cultivation

Under the relevant Danish legislation, remnants of anonymized corneal transplant specimens used for posterior lamellar keratoplasty from donors (aged 30–70) without any corneal disease were obtained from the Danish Cornea Bank (Aarhus University Hospital, Aarhus, Denmark). The specimens were stored in a specific organ-culture storage medium to preserve viability.

The limbus tissues were collected by removing the cornea using a trephine and trimming any remaining tissue from the outer edge. Each limbus was divided in half, dissected into 1–2 mm pieces, and incubated for 1 h at 37 °C in 1 mL of 2 mg/mL collagenase (Roche Diagnostics, United States), for LMSC isolation. The pieces from the second half of the limbus were suspended in dispase (Roche Diagnostics, United States) for an hour at 37 °C to isolate LESC and LM. The resultant cell clusters were collected using reversible cell strainers with a 37 µm pore size. The collected clusters were broken up into single cells by further digestion in 1 mL of 0.25% trypsin and 0.02% EDTA (Gibco, Taastrup, Denmark) at 37 °C for 15 min. For primary cultures, single-cell suspensions were seeded into T25 flasks (Greiner Bio-one, Frickenhausen, Germany) and cultured in a “complete medium” comprising DMEM/F12 (Gibco, Taastrup, Denmark) containing 10% FCS (Gibco, Taastrup, Germany) and 1% penicillin/Streptomycin (Gibco, Taastrup, Denmark) to support LMSCs. Complete media supplemented with 1% Human corneal epithelial supplement (Gibco, Taastrup, Denmark) was used to support LESCs, while complete media supplemented with 1% melanocyte Growth supplement (Sigma Aldrich, Germany) was used to support LM and HEMn (ATCC, Denmark) culture.

The media was changed every other day until the cells reached 80% confluency. Sub-culture was carried out by rinsing the cells twice with 1X sterile PBS (phosphate-buffered saline) (Gibco, Taastrup, Denmark) to remove dead cells and debris before being treated for 90 s with an appropriate amount of TrypLE (Gibco, Taastrup, Denmark) based on the flask size, to detach the cells. The enzyme activity was neutralized by adding media twice the volume of TrypLE, the cell suspension was centrifuged at 500 *g* for 5 min, and the supernatant was removed. The cells were resuspended in the relevant media and transferred to three T75 flasks (Greiner Bio-one, Frickenhausen, Germany). In the second passage, the image of the cells was taken by an inverted microscope (Zeiss, Germany), and their morphology was studied. To remove the contamination with LMSCs, a low concentration of geneticin (0.2 mg/mL) was added to the LM-specific medium for 48 h from passages 1 to 2.

### 2.2 Identification and characterization of isolated cells

Confirmation of the isolated cell types was carried out by flow cytometric characterization of surface and intracellular markers, optimized using the directly labelled antibodies (Table 1). All staining buffers were based on sterile PBS containing 50% Accumax (Sigma-Aldrich) and 25 nM HEPES (Life Technologies) to maintain the appropriate PH range and prevent cell clumping.

**TABLE 1:** Cytometer setup for limbal cell markers.

Markers	Antibody	Fluorochrome	Laser	Emission channel
LMSC markers	CD105	BV510	405 nm	525/40 BP
	CD73	FITC	488 nm	525/40 BP
	CD90	PerCP-Cy5.5	488 nm	690/50 BP
LESC markers	P63	CF488A	488 nm	513/26 BP
	CK3	CF568	561 nm	585/42 BP
LM markers	MITF	CF488A	488 nm	513/26 BP
	TYR	CF568	561 nm	585/42 BP
Viability stain	FVS510		405 nm	525/40 BP
	FVS570		561 nm	585/42 BP

BP: band pass; FVS: fixable viability stain; CF: carboxyfluorescein; BV: brilliant violet; LMSC: limbal mesenchymal stromal cell; LESC: limbal epithelial stem cell; LM: limbal melanocyte.

Dead cells were first eliminated from the analysis following incubation with Fixable Viability Stains 570 (FVS570) and 510 (FVS510) (BD Bioscience, Lyngby, Denmark) (Table 1) at room temperature for 15 min. Positivity thresholds were determined by fluorescence minus one (FMO).

To confirm the presence of LMSCs, the cells were stained with CD90, CD73, and CD105 antibodies (BD Bioscience, Lyngby, Denmark) (LMSC markers) diluted in PBS supplemented with 2% FCS and 0.1% sodium azide (Merck Schuchardt, Hohenbrunn, Germany) at 4 °C for 30 min in the dark.

Cells were fixed and permeabilized to detect intracellular antigens in Fix/Perm buffer (BD Pharmingen, Denmark) containing 5% formaldehyde and 1.76% methanol for 50 min at 4 °C. LESCs were confirmed by staining with P63 (1:100; Biotium, Denmark)) and CK3 (1:200; Biotium, Denmark) antibodies, while LMs were identified using MITF (1:100) (Biotium, Denmark) and Tyrosinase (1:200; Novusbio, USA). All staining steps were incubated for 50 min at 4 °C.

The stained cells were then rinsed and transferred into a 5 mL round-bottom glass FACS tube (BD Falcon, Albertslund, Denmark) for surface epitope analysis using the CytoFLEX (Beckman Colter, Copenhagen, Denmark) flow cytometer. Before analysis, compensation values were established using the BD CompBeads Plus Set Anti-Mouse Ig, κ, and Anti-rat Ig, κ (BD Biosciences, New Jersey, USA). The data were analyzed using Kaluza 2.1 software (Beckman Coulter, Indianapolis, IN, USA), and basic gating was applied to target live singlets, while the top 2.5 percentile of unstained cells (fluorescence minus one (FMO) control) was regarded as positive.

### 2.3 Conditioned media preparation

In the second passage, LESC, LMSC, LM, and HEM cells were first cultured in media containing the respective supplements until they reached 80% confluency. The supplemented medium was then replaced with DMEM/F12 lacking FCS and any other supplements, and were allowed to incubate for 48 h before the supplement-free media was collected and used as CM.

### 2.4 Fibronectin coating

Fibronectin (FN) (Sigma Aldrich, Germany) was diluted in PBS to prepare coating solutions at concentrations of 3, 5, and 8 µg/cm^2^. Three separate 6-well plates were assigned to each concentration. The wells were coated with the respective FN solution, incubated for 1 h at room temperature, and then the coating solution was removed. Wells were subsequently washed once with PBS to remove any unbound material.

A fourth plate, left uncoated, served as a control. All four plates were seeded with LESCs (5 × 10^3^ cells/cm^2^) cultured in complete media containing 1% corneal epithelial growth supplement for 7 days, while the media was refreshed every other day.

### 2.5 Cell proliferation assay

LESCs were seeded at a concentration of 5 × 10^3^ cell/cm^2^ in two sets of 6-well plates, one set containing different concentrations of FN coating (3, 5, and 8 µg/cm^2^) and called FN3, FN5, and FN8 groups, the other set supplemented with LMSC, LM, and HEMn-CM Their proliferation rate was calculated based on the doubling times on days three, five, and seven after the cultivation, as the cells were washed three times with sterile PBS to remove the dead cells or debris and detached using 500 µL TrypLE (Gibco, Taastrup, Denmark). Following a 5-min centrifuge at 500 *g*, the cell suspension was counted using a hemacytometer (Bürker-Türk, Assistant, Germany) under a light microscope (Zeiss, Germany). Doubling time was calculated according to the formula below, where NT was the number of the cells at the end of passage, N0 was the starting number of the cells, T was time in any unit, and doubling time was represented as days:

$$\text{Growth rate }\left(\text{Gr}\right):\;\frac{\mathrm{Ln}\left(\frac{NT}{N0}\right)}{T}$$

Doubling time:ln⁡2/Gr

### 2.6 Real time-qPCR

This part was performed in two steps, as in the first step, we had two sets of four six-well plates. One set was seeded with second passage LESCs (5 × 103/cm^2^) treated with 1:1 complete media containing 1% corneal epithelial growth supplement, and LESC, LMSC-, LM-, or HEM- CM, and one plate was treated with complete media as a control. The second set was coated with previously described concentrations of FN, as FN3, FN5, FN8, and non-coated as a control, and treated with complete media. The cells were cultured for 7 days, and the media was replaced every other day. Based on the results from the initial FN coating and CM supplementation tests, LESC cultures were prepared that combined 3 µg/cm^2^ FN coating and supplementation (1:1) with LM-, LMSC-, and HEMn-CM for 7 days. The cells were then collected and assessed for the expression of *PEDF* and *HES1* markers. In brief, the Aurum Total RNA Mini Kit (Bio-Rad, USA) was utilized for RNA isolation from LESCs at the second passage. The purity and concentration of RNA were determined using a nanodrop spectrophotometer (NanoDrop; Thermo Fisher Scientific, Massachusetts, USA), and first-strand cDNA synthesis was performed using RNA from lysed cultured cells and iScript™ reverse transcriptase kit (Bio-Rad, California, USA). The qPCR reactions were carried out using a CFX Connect Real-Time PCR instrument (Bio-Rad, California, USA), with target-specific primers (TAG Copenhagen A/S, Denmark) (Table 2), IQ SYBR Green Supermix (Bio-Rad, California, USA), and cDNA according to the manufacturer’s instructions. A housekeeping gene, *PPIA* (Peptidylprolyl isomerase A), was used, and *PEDF* (pigment epithelial-derived factor) and *HES1*(Hairy and Enhancer of Split 1) were considered as genes of interest (GOI) to evaluate the role of treatments on proliferative and stemness ability of LESCs. Normalized to *PPIA*, gene expression levels and ratios were compared using the Livak (2^−ΔΔCq^) method, and the Pfaffl method would be accurate when PCR efficiencies are not optimal or differ between target and reference genes.

**TABLE 2:** Primers’ sequences in RT-qPCR.

Gene symbol	Primer sequences	
PPIA	Forward	5′ TCC TGG CAT CTT GTC CAT G 3′
	Reverse	5′ CCA TCC AAC CAC TCA GTC TTG 3′
HES1	Forward	5′ TGG AAA TGA CAG TGA ACC 3′
	Reverse	5′ GTT CAT GCA CTC GCT TTC 3′
PEDF	Forward	5′ TGT GCA GGC TTA GAG GGA CT- 3′
	Reverse	5′ GTT CAC GGG GAC TTT GAA GA - 3′

PPIA: Peptidylprolyl isomerase A; PEDF: pigment epithelial-derived factor; and HES1: Hairy and Enhancer of Split 1.

### 2.7 Statistical analysis

The data were assessed for normal distribution using the Shapiro-Wilks test. The relative expression ratios are reported as the mean fold-change ± standard deviation. Doubling time and changes in fold-regulation of the assessed genes in the different treatment sub-groups were compared using the Kruskal Wallis non-parametric test. All statistical analyses were carried out using the SPSS statistical software (Ver.29; IBM, New York, USA). p < 0.05 was considered as significant and adjusted by Bonferroni correction for multiple tests.

## 3 Results

### 3.1 Cell culture and morphology

**FIGURE 1:**
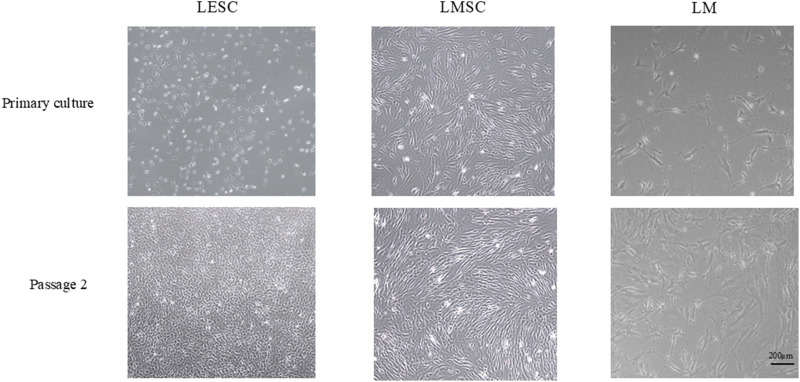
Morphology of limbal niche cell populations in primary culture and at passage 2 (original magnification, 4×). LMSC, limbal mesenchymal stromal cell; LESC, limbal epithelial stem cell; LM, limbal melanocyte.

From passage 1 to 2, the isolated LMSC, LESC, and LM groups demonstrated a mean ± SD cell doubling time of 1.85 ± 0.06, 1.96 ± 0.03, and 2.23 ± 0.25, respectively. The LMSCs presented an elongated or spindle shape with a single nucleus, typical of fibroblasts. LESCs showed a relatively large nucleus compared to the amount of cytoplasm, and LM demonstrated a dendritic morphology characterized by a small cell body with long, branching processes (dendrites) extending outward (Figure 1).

### 3.2 Immunophenotypical characterization of isolated cells

Flow cytometry analysis was conducted to distinguish the various isolated cell populations within the limbal niche (Table 3), demonstrating high expression levels of CD90, CD73, and CD105, confirming the presence of LMSCs. The expression of P63 and CK3, as well as the limbal epithelial cell markers, indicated the presence of LESCs, while the expression of TYR and MITF confirmed the LM population.

**TABLE 3:** Immunophenotypic profiling of isolated populations from the limbal niche.

Cell population	Marker	Marker expression (mean ± SD)
LMSC	CD90	99.48 ± 0.67
	CD73	99.38 ± 0.70
	CD105	89.91 ± 4.70
LESC	P63	64.38 ± 5,40
	CK3	82.20 ± 4.70
LM	MITF	46.49 ± 5.86
	TYR	71.28 ± 8.82

LMSC: limbal mesenchymal stromal cells; LESC: limbal epithelial stem cells; LM: limbal melanocytes.

**FIGURE 2:**
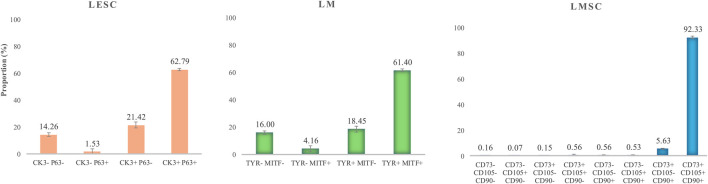
The prevalence of the immunophenotype of limbal niche cells. LESC: limbal epithelial stem cell, LM: limbal melanocyte, LMSC: limbal mesenchymal stromal cell. The data are presented as mean ± standard deviations (SDs).

In the LESC group, 82.12% of the cells presented the CK3 epithelial cell marker, while 64.71% exhibited the P63 stem cell marker, indicating the presence of limbal epithelial stem cells (LESCs). Furthermore, 62.79% of the cells co-expressed both P63 and CK3, indicating that the stemness potency of the limbal epithelial cells is in different stages (Figure 2).

The purity of the isolated cell populations was validated using negative controls; LESCs were confirmed to be negative for CD90 and CD117 expression, LMSCs for CD117 and TYR expression, and LM cells for CD90 and P63.

### 3.3 The impact of conditioned media on LESCs

**FIGURE 3:**
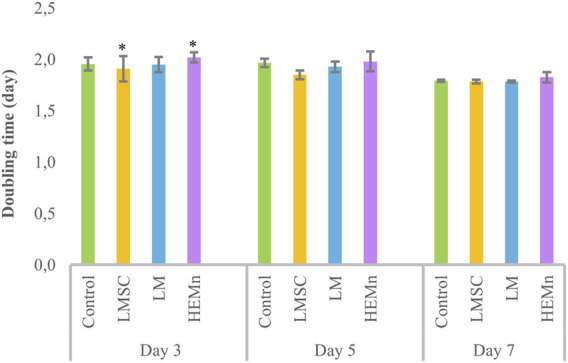
Changes in the doubling time of limbal epithelial stem cells (LESCs) following three, five, and 7 days of treatment with limbal mesenchymal stromal cell (LMSC), limbal melanocyte (LM), and human epidermal melanocyte (HEMn)-derived conditioned media. The data is presented as mean ± standard deviations (SDs). The Pairwise significant differences (p < 0.05) were adjusted for multiple tests using the Bonferroni correction.

Treatment of LESCs with LMSC-derived conditioned media showed a lower doubling time than all other groups, although this difference was only significant (P < 0.05) compared to the HEMn-derived group on day three, and was not pronounced after five and 7 days (Figure 3).

**FIGURE 4:**
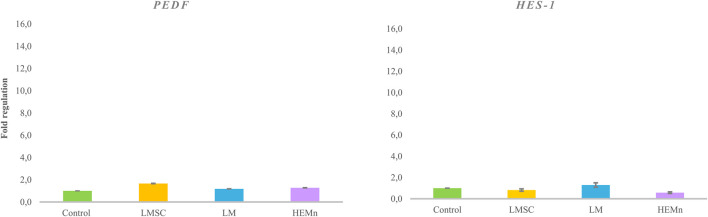
PEDF and HES1 gene expression ratio in limbal epithelial stem cells (LESCs) following treatment of limbal mesenchymal stromal cell (LMSC)-, limbal melanocyte (LM)-, and human epidermal melanocyte (HEMn)-derived conditioned media, normalized to non-treated LESCs. The data is presented as mean ± standard deviations (SDs).

Supplementation with LMSC, LM, and HEMn-conditioned media did not result in statistically significant changes in *PEDF* or *HES1* expression overall. Meanwhile, conditioned media from LMSC and LM exhibited a trend toward increased expression of *PEDF* (1.5-fold) and *HES1* (1.29-fold), respectively (Figure 4).

### 3.4 Effect of fibronectin coating on LESCs

**FIGURE 5:**
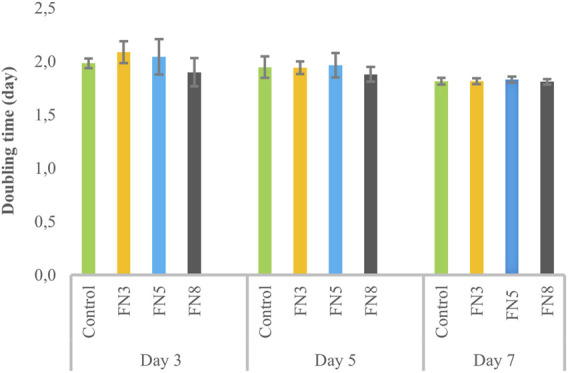
Effect of coating with various fibronectin (FN) concentrations on the proliferation ability of limbal epithelial stem cells (LESCs). The data is presented as mean ± standard deviations (SDs). The Pairwise significant differences (p < 0.05) were adjusted for multiple tests using the Bonferroni correction.

**FIGURE 6:**
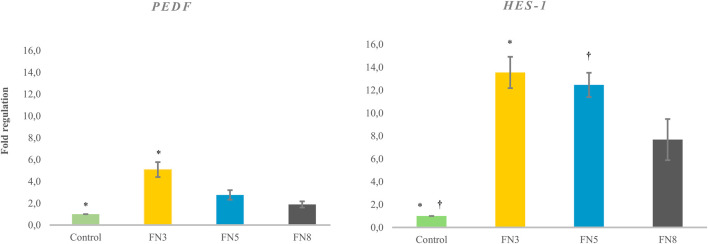
Effect of coating with different fibronectin (FN) concentrations on the stemness and self-renewal ability of limbal epithelial stem cells (LESCs). The data is presented as mean ± standard deviations (SDs). Pairwise significant differences (P < 0.05) in each row are indicated with * and † and adjusted by the Bonferroni correction for multiple tests.

Coating with different concentrations of FN did not show any significant effect on the proliferation ability of LESCs (Figure 5). However, FN coating at a concentration of 3 µg/cm^2^ resulted in a 5.09 (±0.685)-fold upregulation of *PEDF* gene relative to the control (p < 0.05). FN coating at concentrations of 5 and 8 µg/cm^2^ exhibited lower *PEDF* expression than 3 µg/cm^2^, while still higher than the control, although non-significant (Figure 6).

FN coatings at 3 µg/cm^2^ and 5 µg/cm^2^ presented a 13.5 (±1.370)- and 12.4 (±1.061)- fold higher HES1 gene expression, respectively, compared with the control group (p < 0.05). FN coating at 8 µg/cm^2^ concentrations showed a lower *HES1* expression than 3, and 5 µg/cm^2^, while still higher than the control, although non-significant (Figure 6).

### 3.5 Effect of fibronectin and conditioned media combination on LESCs

**FIGURE 7:**
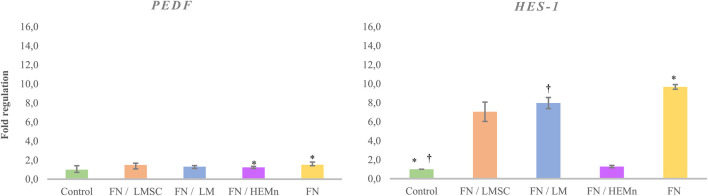
Expression of PEDF and HES1 genes in limbal epithelial stem cells (LESCs) after 7 days of culture on the 3 µg/cm^2^ fibronectin (FN) alone or combined with conditioned media from limbal mesenchymal stromal cell (LMSC), limbal melanocyte (LM), and human epidermal melanocyte (HEMn) culture. The data is presented as mean ± standard deviations (SDs). The Pairwise significant differences (p < 0.05) were adjusted for multiple tests using the Bonferroni correction.

While coating with 3 µg/cm^2^ FN resulted in a significant upregulation of *PEDF* and *HES1,* adding limbal niche cell-derived conditioned media alongside this FN concentration did not further enhance PEDF upregulation compared to the control group (p < 0.05). However, the results showed that FN coating alone led to a 3.28 (±0.283)-fold increase in *PEDF* gene expression; this improvement was higher compared to the 1.03 (±0.283)-fold change observed with FN coating supplemented with HEMn-CM (p < 0.05). The only FN-coated and combining FN-coating supplemented with LM-CM groups indicated 9.67 (±0.247) and 7.59 (±1,584)-fold upregulation of *HES1,* respectively, compared to the control group (p < 0.05). At the same time, HEMn-CM demonstrated results similar to those of the non-treated control group (Figure 7).

## 4 Discussion

Corneal transparency depends on the normal function of the LESCs and their interactions with limbal niche cells and extracellular matrix (ECM) (Robertson et al., 2021; Bonnet et al., 2021). While proliferating is a primary and essential characteristic of stem cells (Kulkarni et al., 2010; Robertson et al., 2021), remaining quiescent, characterized by a non-proliferative state, is also vital for the long-term maintenance of adult stem cells and tissue homeostasis (Urbán et al., 2019). The limbal niche regulates the balance between proliferation and differentiation, preserving quiescence while maintaining stemness potential (Urbán et al., 2019; Robertson et al., 2021; Bonnet et al., 2021). This allows LESCs to remain dormant until paracrine factors trigger proliferation and regeneration when repair is needed (de Morree and Rando, 2023; Urbaìn and Cheung, 2021). This study aimed to independently assess the potential effects of limbal niche cells’ conditioned media, which may provide limbal paracrine factors, and Fibronectin (FN) coating as one of the ECM components, on the proliferation, quiescence, and stemness of LESCs *in vitro*. Considering the suggested influence of pigmentation on the LESCs’ stemness (Liu et al., 2018), human epidermal melanocytes (HEMn) were included as a comparator to limbal niche melanocytes, to investigate the potential niche-specific interaction of these cells (Upadhyay et al., 2021; Li et al., 2006). None of the conditioned media showed a considerable difference in the proliferation ability or stemness of LESCs compared to the control group, while FN coating significantly enhanced stemness and self-renewal ability without impairing their proliferation ability.

Previous studies have demonstrated that different limbal niche cells, including LESCs, could be isolated from limbal tissue using enzymatic digestion followed by culture in cell-type-specific supplemented media (Polisetti et al., 2022; Dziasko et al., 2015). In this study, the identity of LMSC, LESC, and LM isolated from the limbal tissue was confirmed by their morphological characteristics (Polisetti et al., 2022; Dziasko and Daniels, 2016; Dziasko et al., 2014; Polisetti et al., 2020) and expression of cell-type-specific immunophenotypic markers (Dziasko and Daniels, 2016; Dziasko et al., 2014; Polisetti et al., 2020; Cui and Man, 2023).

Conditioned medium from *in vitro* culture contains the cell secretions and can act as a reservoir of growth factors, facilitating regeneration (Jabbehdari et al., 2020a; Osugi et al., 2012; Smolinská et al., 2023; Jabbehdari et al., 2020b). Several animal studies have shown that conditioned media, whether from uterine cervical stem cells (Bermudez et al., 2015), corneal mesenchymal stromal cells (Jabbehdari et al., 2020a), or LMSCs (Amirjamshidi et al., 2011), can accelerate corneal wound healing *in vivo* by delivering growth factors and modulating inflammation. This effect may involve IL-1–induced upregulation of hepatocyte growth factors (HGF) and keratinocyte growth factor (KGF) in stromal cells, promoting proliferation, migration, and transition from the inflammatory to the proliferative phase (Wilson, 2020).

LMSCs are mesenchymal stromal cells that can produce and release various growth factors that increase cell proliferation (Zhuang et al., 2021; Hefka Blahnova et al., 2020). An *in vivo* study on an animal model of limbal stem cell deficiency (LSCD) showed that topical treatment with conditioned media obtained from limbal fibroblasts (mesenchymal stromal cells) enhanced the growth of corneal epithelium, while skin fibroblast-derived conditioned media supported the growth of conjunctival type epithelium in the same model, suggesting that this proliferative effect may be niche-specific (Amirjamshidi et al., 2011). Another previous study demonstrated the effectiveness of HEMn-derived conditioned media in promoting keratinocyte proliferation *in vitro* (Deveci et al., 2001).

In the present study, LMSC-conditioned media showed a significantly enhanced proliferation rate of LESCs compared to HEMn-derived conditioned media, supporting the niche-specific nature of paracrine signaling. These effects appear dependent on the cellular origin and local microenvironment, where LMSC-secreted factors promote proliferation, while other niche components may usually help preserve quiescence (Urbán et al., 2019; Polisetti et al., 2021; Li et al., 2006; Bermudez et al., 2015).

However, LMSC-conditioned media did not significantly impact LESC proliferation rates, which could be attributed to the *in-vitro* model lacking the cascade of proinflammatory cytokines released during cell damage *in vivo* (Weng et al., 1997; Xiao et al., 2020). Furthermore, preparing conditioned media under serum-free conditions may induce stress-related alterations in the secretum, and potentially affecting its content (Jin et al., 2022). This represents a limitation of the current study, as serum-free conditions may not fully replicate the native paracrine environment.

The conditioned media failed to create an optimal environment to maintain quiescence, characterized by a non-proliferative state (de Morree and Rando, 2023; Urbán et al., 2019), as they could not significantly enhance the upregulation of *PEDF* and *HES1* relative to controls. However, LMSC-derived growth factors and LM relatively increased *PEDF* and *HES1*gene expressions, respectively. Liu et al. (2018) suggested a link between pigmentation and the stemness potential of LESCs. This pigmentation was attributed to melanocytes dispersed within the basal epithelium of the limbus, which could potentially enhance the stemness of LESCs (Liu et al., 2018; Polisetti et al., 2021). In our study, however, HEMn-derived conditioned media did not improve stemness. Whereas LM-conditioned media upregulated *HES1* expression, suggesting that limbal melanocytes promote self-renewal through niche-specific mechanisms beyond pigmentation. This aligns with evidence that melanocytes from different niches exhibit distinct properties shaped by their developmental origins and microenvironments (Liu et al., 2018; Polisetti et al., 2021; Zocco and Blanpain, 2017). Thus, limbal melanocytes appear more effective than epidermal melanocytes in supporting the quiescent state of LESCs.

The direct interaction between stem cells and the ECM plays a significant role in their proliferative or quiescent state (de Morree and Rando, 2023). As a primary component of the limbal niche ECM, FN closely interacts with LESCs and can improve their self-renewal ability through the Wnt non-canonical pathway (Robertson et al., 2021; Zheng et al., 2019). The findings of the current investigation align with previous studies, indicating that FN can promote LESC stemness and self-renewal ability by upregulating *PEDF* and *HES1* gene expression. However, despite varying FN concentrations, this glycoprotein did not enhance the proliferation rate of LESCs based on their doubling time.

Different signalling pathways, including canonical and non-canonical Wnt and Notch, regulate LESC fate and maintenance (Robertson et al., 2021). Notch signalling, via its downstream effector *HES1*, is strongly expressed in the limbal epithelium and is central to maintaining a reserve of quiescent stem cells for corneal regeneration (Kulkarni et al., 2010; Mikhailova et al., 2015; Ahmadi and Jakobiec, 2002; Djalilian et al., 2008). In this study, FN upregulated *HES1* expression without altering proliferation, suggesting that FN may promote self-renewal by engaging Notch-related mechanisms, though the downstream interactions remain to be clarified and validated in future mechanistic studies. The upregulation of *HES1 in vitro* may therefore enhance the self-renewal capacity of LESCs in their quiescent state, even in the absence of a proliferative response, as observed when LESCs were in direct contact with FN in the present study (Yu et al., 2010; Giannasi et al., 2023; Cichorek et al., 2013; Nakamura et al., 2008).

*PEDF* has been shown to enhance the regeneration of the cornea and limbus in animal models (Cichorek et al., 2013; Yeh et al., 2015) through activating signalling pathways, such as MAPK and STAT, which are essential for cell proliferation (Yu et al., 2010; Giannasi et al., 2023; Fan et al., 2019). The preliminary findings are consistent with previous reports, suggesting that FN may promote LESC stemness and self-renewal by upregulating *PEDF* and *HES1*, without increasing proliferation.

## 5 Conclusion

This preliminary study suggests that FN coating generally upregulated the expression of *PEDF* and *HES1* genes, with this effect being most prominent at 3 µg/cm^2^. It significantly increased *PEDF* expression, but this effect was diminished by conditioned media, highlighting FN’s primary role over paracrine factors in promoting LESC self-renewal. Conversely, FN coating enhanced *HES1* expression, further improved with LM-derived conditioned media, indicating a combined role of FN and paracrine factors in regulating LESC self-renewal via *HES1*. These exploratory findings raise the possibility of utilizing niche-cell-conditioned media and direct contact with FN on the self-renewal ability of LESCs *in vitro*. Further mechanistic and functional studies are required to validate and expand upon these preliminary observations.

## Data Availability

The data that support the findings of this study are available from the corresponding author (SA), upon reasonable request.
